# Health Outcomes in Acromegaly: Depression and Anxiety are Promising Targets for Improving Reduced Quality of Life

**DOI:** 10.3389/fendo.2014.00229

**Published:** 2015-01-06

**Authors:** Victor Jacobus Geraedts, Christina Dimopoulou, Matthias Auer, Jochen Schopohl, Günter Karl Stalla, Caroline Sievers

**Affiliations:** ^1^Max Planck Institute of Psychiatry, Munich, Germany; ^2^Leiden University Medical Center, Leiden, Netherlands; ^3^Medizinische Klinik Innenstadt, Ludwig-Maximilians University, Munich, Germany

**Keywords:** acromegaly, anxiety, biochemical control, depression, pituitary adenomas, quality of life

## Abstract

**Introduction:** Remission criteria of acromegaly are based on biochemical variables, i.e., normalization of increased hormone levels. However, the established reduction in Quality of Life (QoL) is suggested to be independent of biochemical control. The aim of this study was to test which aspects predict QoL best in acromegaly.

**Methods/design:** This is a prospective cohort study in 80 acromegalic patients, with a cross-sectional and longitudinal part. The main outcome measure was health-related QoL, measured by a generic and a disease-specific questionnaire (the SF-36 and AcroQoL). Main predictors were age, gender, biochemical control, disease characteristics, treatment modalities, and psychopathology.

**Results:** Our cohort of 80 acromegalics had a mean age 54.7 ± 12.3 years with an average disease duration of 10.8 ± 10.0 years. Ratio macro-/microadenoma was 54/26. In adjusted mixed method models, we found that psychopathology significantly predicts QoL in acromegaly (in models including the variables age, gender, disease duration, tumor size, basal hormone levels, relevant treatment modalities, and relevant comorbidities), with a higher degree of psychopathology indicating a lower QoL (depression vs. AcroQoL: *B* = −1.175, *p* < 0.001, depression vs. SF-36: *B* = −1.648, *p* < 0.001, anxiety vs. AcroQoL: *B* = −0.399, *p* < 0.001, anxiety vs. SF-36: *B* = −0.661, *p* < 0.001). The explained variances demonstrate superiority of psychopathology over biochemical control and other variables in predicting QoL in our models.

**Discussion:** Superiority of psychopathology over biochemical control calls for a more extensive approach regarding diagnosing depression and anxiety in pituitary adenomas to improve QoL. Depressive symptoms and anxiety are modifiable factors that might provide valuable targets for possible future treatment interventions.

## Introduction

Objectives for health outcomes include reduction of mortality, morbidity, and the improvement of Quality of Life (QoL) (1).

However, in patients with pituitary adenomas, such as acromegaly, this third treatment goal remains often unfulfilled as they report to have a markedly reduced QoL, which often persists under biochemical control/remission (2–7).

Various factors have been suggested to be associated with reduced QoL in pituitary disease, particularly acromegaly; however, there is no clear consensus in this regard.

The current consensus criteria for cure and remission of acromegaly are based on biochemical variables, i.e., normalization of elevated hormonal levels of the biomarkers growth hormone (GH) and/or insulin like growth factor-1 (IGF-1) (8–10). However, the definition of such a “remission” remains insufficient with regard to absolute recovery: a drawback of a purely biochemical approach is that there are strong indications that biochemical control does not necessarily provide complete “cure” in patients’ view, since their health-related QoL remains reduced in most patients with acromegaly.

However, there is no consensus on the value of these contributing factors. For instance, several articles reported biochemical control to have no significant association with reduced QoL (11–13), whereas other articles report exactly the opposite (14–16), indicating a clear need for elucidation.

Psychopathological variables are candidate modifiable factors to link pituitary disease, especially acromegaly, to a lower health-related QoL. On the one hand, acromegaly is reported to be associated with neuropsychiatric comorbidities such as depressive symptoms (17) and anxiety (5, 18, 19). On the other hand, there is a clear association between psychopathology and perceived QoL (20–22).

The aim of this study was therefore to test the predictive impact of psychopathology (depressive symptoms and anxiety) on health-related QoL in acromegalic patients.

For this purpose, a theoretical model (23) was applied on acromegaly and tested in two primary data sets: (a) a cross-sectional cohort of 80 acromegalic patients, which was (b) subsequently validated in the longitudinal cohort of the same patients.

## Materials and Methods

### Study design

This is a prospective cohort study including two analytic parts: a cross-sectional and prospective evaluation. For design and recruitment of the initial cross-sectional cohort, see previous publications from our group (18, 19). Six years after the cross-sectional evaluation (baseline), a longitudinal evaluation was performed in which the same patients were recontacted and asked to participate using the same diagnostic instruments.

### Study participants

For the cross-sectional analyses, 80 acromegalic patients (response rate 56%) were included. Patients were recruited at the Endocrine Outpatient Clinic at the Max Planck Institute of Psychiatry and the Medizinische Klinik und Poliklinik IV, Ludwig-Maximilians-Universität Munich.

For the longitudinal analyses, patients were recontacted and recruited accordingly. At both timepoints, patients were contacted by a letter regarding the aim and design of the study. A further request for participation was issued via telephone for initial non-responders.

Thirty-six acromegalic patients (response rate 45%), were included at the follow-up timepoint.

The project was approved by the medical ethics committee of the Ludwig-Maximilians-Universität Munich; all patients gave their written informed consent.

### Measurement instruments

#### Clinical characteristics

Patients were given questionnaires with standardized psychometric instruments, which allowed assessment of disease-related variables, therapy history, symptoms, tumor characteristics, comorbidities and current complaints, and evaluation of psychopathological symptoms. Patients were seen either at the Max Planck Institute for Psychiatry or at the Ludwig-Maximilians-Universität Munich for a standardized clinical assessment, which included a physical examination and laboratory analyses. Additional information was retrieved from the patient files if necessary.

#### Laboratory measurements

Biochemical control (dichotomous classification) was based on a single serum sample of patients with confirmed disease. Biochemical control of acromegaly was defined as (1) GH levels <1 μg/l during a glucose-tolerance test over 2 h and (2) IGF-1 levels within 2 SD of an age- and gender-adjusted standardized sample (8).

Pituitary function was routinely assessed in all patients on a yearly basis, with basal fasting measurements of IGF-1, thyroid stimulating hormone (TSH), free thyroxine, total triiodothyronine, luteinizing hormone (LH), follicle-stimulating hormone (FSH), prolactin, and testosterone (men) or estradiol (female). Moreover, stimulation tests were administered including short ACTH test, GHRH/arginine test, and insulin hypoglycemia test, if indicated. All patients were studied under optimal replacement therapy (24).

#### Neuropsychiatric assessment

Quality of Life was measured using the specifically designed instrument AcroQoL (range 0–110) (25), and the general instrument SF-36 (range 0–100) (26, 27). All QoL instruments were arranged to have higher scores reflecting a better QoL.

The following neuropsychiatric variables were assessed: depressive symptoms [Becks Depression Inventory (BDI) (28), range 0–63] and anxiety [State-Trait Anxiety Inventory (STAI) (29), range 40–160]. Neuropsychiatric scoring-instruments were arranged to have higher scores reflect greater disability.

All questionnaires were self-completed.

### Statistical analysis

All analyses were performed with the Statistical Package for the Social Sciences 20.0 Software (SPSS 20.0).

### Descriptive analyses

Differences between biochemically controlled and uncontrolled patients for demographic variables were analyzed using χ^2^-tests and independent samples *t*-tests. Significance was set at the 0.05 level.

### Statistical models

Six-Block linear regression analyses were carried out to assess the contribution of different variables to the outcome QoL. Block 1 contained age and gender, block 2 contained tumor size (dichotomous: macro- vs. microadenoma), basal GH levels, and disease duration, block 3 contained treatment types [dichotomous: surgery, radiation therapy, octreotide, lanreotide, dopamine-agonists (bromocriptin, lisuride, cabergoline, quinagolide), pegvisomant], block 4 contained comorbidities (dichotomous: arrhythmia, cardiomyopathy, cerebrovascular diseases, arterial hypertension, coronary artery disease, history of myocardial infarction, arthralgia, arthropathy, carpal tunnel syndrome, diabetes mellitus type 2, pathological glucose-tolerance, pituitary insufficiency, sleep apnea, lung diseases, cancer), and block 5 contained the psychopathological variables (depressive symptoms, anxiety). Separate analyses were carried out for depressive symptoms and anxiety (block 5) due to large correlations (Pearson’s *r* = 0.713, *p* < 0.001). Block 6 contained the variable biochemical control as a predictor. A forced entry method was used for block 1, 2, 5, and 6 as we deemed inclusion of these variables, a basic requirement for our model. A stepwise forward likelihood ratio method was used for blocks 3 and 4 to include only those predictors that carried significant predictive value. The likelihood ratio method is preferable over the other stepwise methods (30).

For the longitudinal analyses to determine predictors of long-term QoL, linear mixed-effect models with a first-order autoregressive covariance matrix for repeated effects were used. Linear mixed-effects models allow a flexible length of follow-up for separate patients and account for within-patient variations (31, 32). Separate regression coefficients and intercepts were created for each individual patient. To investigate the influence of psychopathology on progression of QoL, the interaction between time and depressive symptoms/anxiety was investigated. A similar approach was taken to investigate if biochemically controlled patients progress differently throughout time in terms of QoL than their uncontrolled counterparts by investigating an interaction between time and biochemical control. Variables that yielded a significant contribution in the baseline model were implemented as factors/covariates in all longitudinal models, as well as age, gender, disease duration, and basal hormonal levels, to account for confounding. Normality was confirmed by examining normal probability plots. Significance was set at the 0.05 level.

### Missing values

If 15% or more of the data from a questionnaire or scale was missing, data from that variable was excluded from analysis. Remaining missing values were filled in according to the corresponding scoring instructions or by using the median from the separate items if the scoring instructions lacked a suggestion for dealing with missing values. An exception was made for the covariate biochemical control; missing data in this domain resulted in exclusion from analysis for that patient.

## Results

### Demographic and clinical characteristics

Mean age for the total acromegalic cohort was 54.7 ± 12.3 years with an average disease duration of 10.8 ± 10.0 years. Fifty-four patients had a macroadenoma, 26 patients had a microadenoma. At baseline, 31 patients were biochemically uncontrolled, and 49 patients were biochemically controlled. Mean basal hormonal level was 2.8 ± 5.0 μg/l for GH and 214.2 ± 161.2 nmol/l, for IGF-1.

For the follow-up analyses, 36 patients participated, mean follow-up time was 7.1 ± 0.7 years – 15 patients remained uncontrolled, whereas 21 patients were biochemically controlled at follow-up. Demographic and clinical characteristics (cross-sectional and longitudinal) are shown in Table 1.

**Table 1:** **Demographic variables according to disease control**.

	Uncontrolled	Controlled	P*
Subjects at baseline (*N*)	80		
	31	49	
Subjects at follow-up	36		
	15	21	
Follow-up time (years)^a^	7.1 ± 0.7		
	7.1 ± 0.5	7.1 ± 0.8	1.000
Age (years)^a^	54.7 ± 12.3		
	51.9 ± 14.0	56.7 ± 10.8	0.088
Disease duration (years)^a^	10.8 ± 10.0		
	10.6 ± 10.6	11.2 ± 9.6	0.822
% Male^b^	46.3 (37)		
	54.8 (17)	40.8 (20)	0.220
% Macroadenoma^b^	68.8 (55)		
	80.6 (25)	61.2 (30)	0.068
Basal GH^c^	2.8 ± 5.0		
	5.3 ± 7.1	1.1 ± 0.9	0.003

*p-Values computed using independent *t*-tests, chi square tests for the variables % Male and % Macroadenoma.

*^a^Mean ± SD*,

*^b^% (*N*)*.

*^c^Basal hormonal levels: serum GH for acromegaly (μg/l)*.

### Association between psychopathology/biochemical control and QoL at baseline

At baseline, our analyses demonstrate that depressive symptoms significantly predicted QoL measured by AcroQoL (*p* < 0.001) and SF-36 (*p* < 0.001) in acromegaly in a model including age, gender, disease duration, basal GH levels, tumor size, and possible treatment modalities and comorbidities as covariates. Negative coefficients reflect a greater amount of depressive symptoms to be indicative of a greater impairment of QoL. The amount of variance explained (ΔR^2^) by depressive symptoms is 0.261 for the AcroQoL and 0.285 for the SF-36, which was the highest variance among all covariates in models explaining a variance (*R*^2^) of 0.637 (AcroQoL) and 0.577 (SF-36).

Similarly, results demonstrate anxiety to be significantly predictive for the AcroQoL (*p* < 0.001) and the SF-36 (*p* < 0.001) in acromegaly. Negative coefficients reflect a greater amount of anxiety to be indicative of a greater impairment of QoL. Δ*R*^2^ is 0.149 for AcroQoL and 0.256 for SF-36 in models with an *R*^2^ of 0.505 (AcroQoL) and 0.543 (SF-36).

Biochemical control was not significantly associated with QoL in acromegaly.

**Figure 1:**
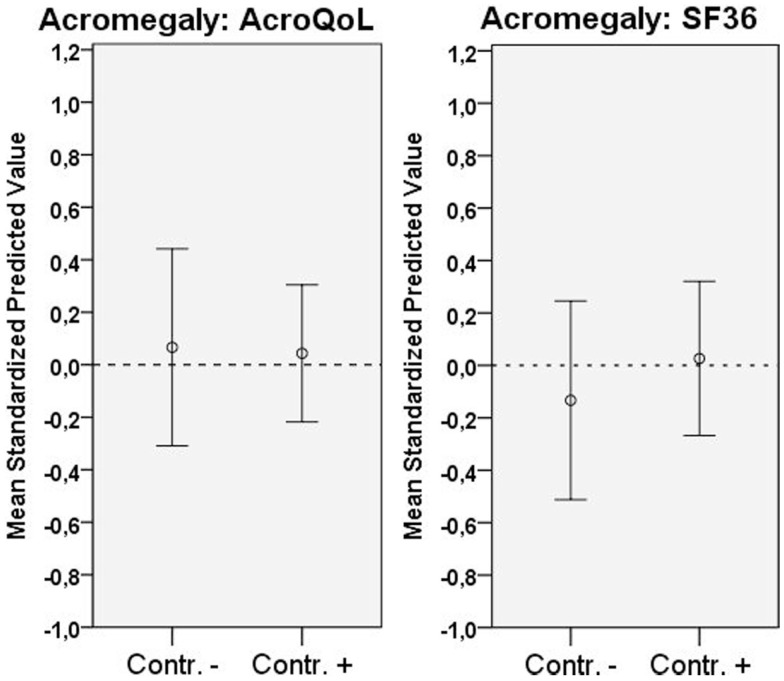
**Distribution of QoL according to scale (mean standardized predicted values), and biochemical status**. Error bars represent 95% CI.

**Figure 2:**
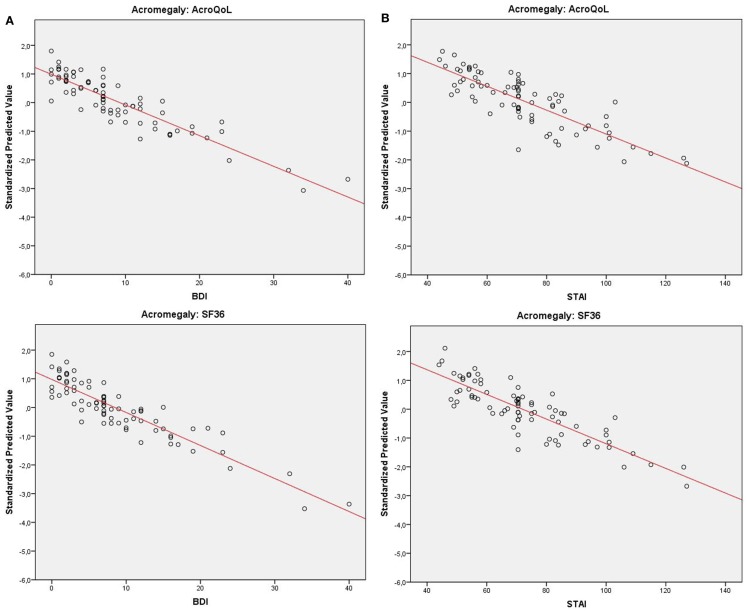
**(A)** Distribution of QoL in acromegaly (standardized predicted values) relative to depressive symptoms with fitted trend-line. **(B)** Distribution of QoL in acromegaly (standardized predicted values) relative to anxiety with fitted trend-line.

Coefficients, *p*-values, and explained variances (*R*^2^) are shown in Table 2, Figure 1 (biochemical control), and Figure 2 (psychopathology).

**Table 2:** **Association of psychopathology/biochemical control and QoL at baseline**.

Disease (scale)	Variable	*B* (SE)	p*	Δ*R*^2^ after correction^a^	Model *R*^2^
Acromegaly (AcroQoL)^b^	**Depressive symptoms**	**−1.175 (0.170)**	**<0.001**	**0.256**	0.688
	Biochemical control	**−**1.157 (3.009)	0.702	0.001	
	**Anxiety**	**−0.399 (0.089)**	**<0.001**	**0.147**	0.578
	Biochemical control	**−**0.546 (3.497)	0.876	<0.001	
Acromegaly (SF-36)^c^	**Depressive symptoms**	**−1.648 (0.256)**	**<0.001**	**0.279**	0.618
	Biochemical control	2.724 (4.497)	0.547	0.002	
	**Anxiety**	**−0.661 (0.109)**	**<0.001**	**0.258**	0.598
	Biochemical control	3.126 (4.614)	0.501	0.003	

*p-Values were computed using linear regression.

*^a^All models carried a correction for age, gender, disease duration, basal hormone levels and tumor size*.

Additional correction for:

*^b^radiation, pathological glucose intolerance and arthralgia*,

*^c^radiation, arthralgia*.

*Bold font marks those results with statistical significance*.

### Association between psychopathology/biochemical control and QoL at follow-up

At follow-up (longitudinal analyses), results demonstrate depressive symptoms to be significantly predictive of QoL measured by AcroQoL (*p* < 0.001) and SF-36 (*p* = 0.001) in acromegaly in a similar model with age, gender, disease duration, basal GH levels, tumor size, and possible treatment modalities and comorbidities as covariates. Similarly, results demonstrate anxiety to be predictive of QoL measured by AcroQoL (*p* < 0.001) and SF-36 (*p* < 0.001) in acromegaly. Negative estimates reflect a greater amount of depressive symptoms/anxiety to be indicative of impaired QoL.

Biochemical control was otherwise not significantly predictive of QoL in other analyses.

Estimates and *p*-values are shown in Table 3.

**Table 3:** **Association of psychopathology/biochemical control and QoL at follow-up**.

Disease (scale)	Variable	Estimate (SE)^a^	P*
Acromegaly (AcroQoL)^b^	**Depressive symptoms**	**−1.213 (0.154)**	**<0.001**
	Biochemical control	**−**1.973 (2.910)	0.500
	**Anxiety**	**−0.405 (0.074)**	**<0.001**
	Biochemical control	**−**1.281 (3.473)	0.713
Acromegaly (SF-36)^c^	**Depressive symptoms**	**−1.601 (0.236)**	**0.001**
	Biochemical control	2.420 (4.072)	0.555
	**Anxiety**	**−0.666 (0.087)**	**<0.001**
	Biochemical control	3.647 (4.471)	0.418

*p-Values computed using linear mixed models.

*^a^All models carried a correction for age, gender, disease duration, basal hormone levels, and tumor size*.

Additional correction for:

*^b^radiation, pathological glucose intolerance and arthralgia*,

*^c^radiation, arthralgia*.

*Bold font marks those results with statistical significance*.

### Association between psychopathology/biochemical control and progression of QoL throughout time

**Figure 3:**
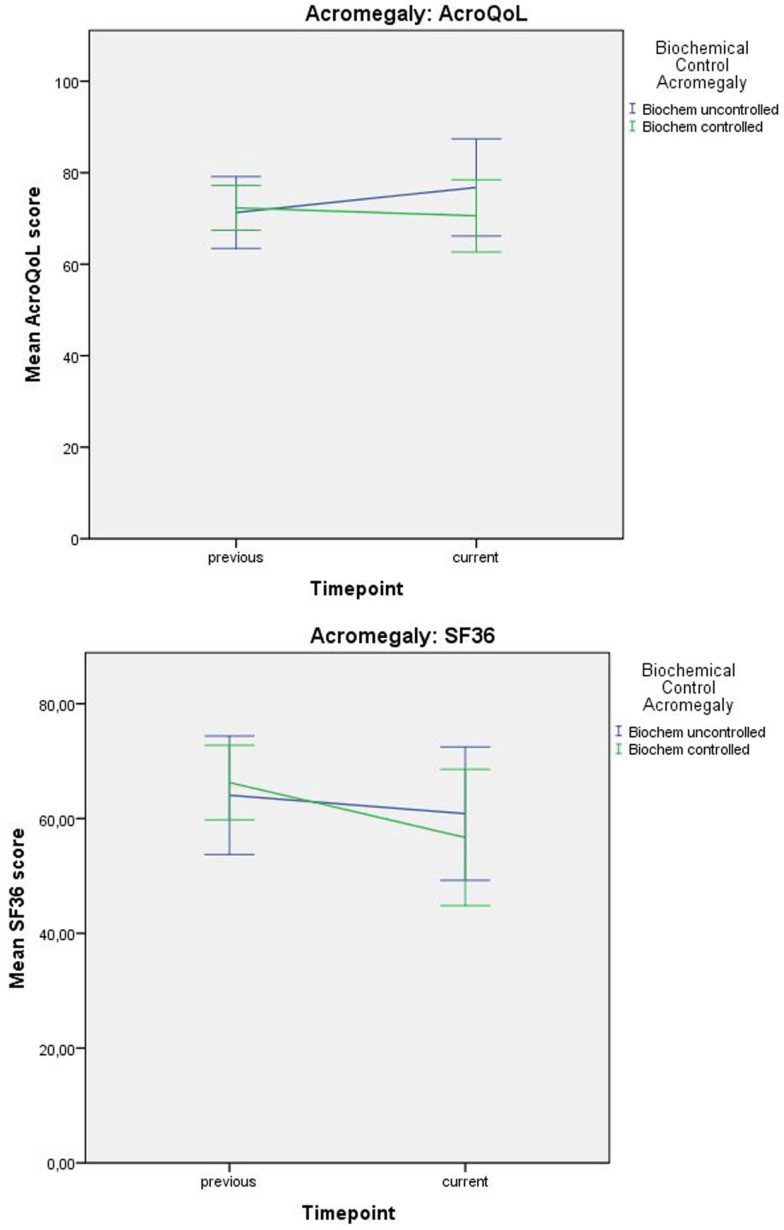
**Progression of QoL (mean values) in acromegaly relative to biochemical status**. Error bars represent 95% CI.

Interaction between time and depressive symptoms, time and anxiety, and time and biochemical control was not significantly associated with QoL in acromegaly, indicating no influence of depressive symptoms, anxiety, or biochemical control on the progression of QoL throughout time. Estimates and *p*-values are shown in Table 4 and Figure 3.

**Table 4:** **Association of psychopathology/biochemical control and progression of QoL**.

Disease (scale)	Variable	Estimate (SE)^a^	P*
Acromegaly (AcroQoL)^b^	Depressive symptoms*time	**−**0.107 (0.222)	0.632
	Biochemical control*time	0.272 (3.244)	0.934
	Anxiety*time	**−**0.005 (0.041)	0.900
	Biochemical control*time	0.434 (3.743)	0.908
Acromegaly (SF-36)^c^	Depressive symptoms*time	**−**0.121 (0.335)	0.922
	Biochemical control*time	**−**1.512 (4.823)	0.926
	Anxiety*time	**−**0.052 (0.047)	0.274
	Biochemical control*time	**−**1.712 (4.271)	0.691

*p-Values computed using linear mixed models.

*^a^All models carried a correction for age, gender, disease duration, basal hormone levels and tumor size*.

Additional correction for:

*^b^radiation, pathological glucose intolerance, and arthralgia*,

*^c^radiation, arthralgia*.

## Discussion

The aim of this study was to analyze the relative impact of previously described predictors in relation to psychopathology on the patient-related health outcome “QoL” in acromegaly.

Since most of the known variables associated with a poor health-related QoL in acromegaly are not modifiable (such as age, gender, disease duration and tumor size), we focused on psychopathology, which would reflect a treatable component that is commonly underdiagnosed.

Although the relationship between QoL and psychopathology has been described before (20–22) in parallel to a solid recognition of reduced QoL in pituitary adenomas and acromegaly (2, 3), we found that the marked reduction of QoL is driven dominantly by psychopathology rather than biochemical control or other factors, which is new information. A potential reason for this observation is that hormonal factors in acromegaly are not necessarily noticeable on a daily basis whereas psychopathological factors are much more dominantly present. The overbearing character of psychopathology may therefore exert a larger influence on a patient’s QoL than the less obvious biochemical control.

It has been previously described that psychopathology is an independent predictor of QoL rather than a masked way of measuring QoL in pituitary patients (6). The scientific implications of our research are augmented by the World Health Organization, which has long recognized the crucial role of QoL in patient-oriented clinical approaches (33). Moreover, a clear association between high QoL scores and a longer survival duration in cancer patients has been described previously (34). These results are in agreement with an earlier study that argues the importance of adding a QoL component rather than sole biochemical considerations in order to improve patient management (35). The findings that psychopathology, rather than biochemical control, drives reduced QoL based on the demonstrated explained variances complements the well-recognized reduction of QoL in pituitary adenomas and leads to our recommendation to place greater emphasis on the role of psychopathology in acromegaly.

Aside from scientific implications, key in the clinical application of this research is the finding that important predictors of reduced QoL are depressive symptoms and anxiety, which are essentially modifiable predictors. A more complex treatment strategy including a more extensive psychopathological evaluation and therapy may be an attractive possibility to improve patient management in pituitary adenomas and especially acromegaly.

### Strengths and limitations

Strengths of our study are the longitudinal design, the two-center approach, the large amount of potential confounders that are accounted for and the usage of validated and disease-specific questionnaires. Furthermore, our study yields new and additional information to expand on previous research and has obvious clinical relevance.

Limitations of our study as in each and every longitudinal study are the potential bias that is introduced due to a preferential “loss-to-follow-up.” Reasons for this non-response were, e.g., disinterest to participate in a large questionnaire, feeling of being cured, and associated disinterest to participate in medical research, deterioration, and associated inability to fill out questionnaires or death. The reasons for non-compliance in our study were not systematically studied. Additionally, not all potential influential factors have been included in the study, e.g., no available data on anti-depressant drug-usage, which may be influential on the severity of the depressive symptoms.

Future research should focus on improving the response rates and validate the observed findings in preferably larger cohorts. Systematic reviews of the literature should attest whether there are other (ideally modifiable) predictors of QoL in acromegaly aside from psychopathology to identify multiple targets for improving QoL (research in progress). Trials with modifying these variables could ultimately verify their clinical applicability (protocol submitted for ethical vote, phase 4 trial EudraCT 2014-000265-43).

## Conclusion

Results indicate biochemical control of acromegaly to be unassociated with both generic and disease-specific QoL. Psychopathology seems to predominantly drive reduced QoL in acromegaly. Hence, we recommend scrutinous systematic screening for psychopathology leading to subsequent specific therapy in acromegaly to test the effect on improving QoL.

## Conflict of Interest Statement

The authors declare that the research was conducted in the absence of any commercial or financial relationships that could be construed as a potential conflict of interest.
